# Psychometric properties of a test in evidence based practice: the Spanish version of the Fresno test

**DOI:** 10.1186/1472-6920-10-45

**Published:** 2010-06-16

**Authors:** Josep M Argimon-Pallàs, Gemma Flores-Mateo, Josep Jiménez-Villa, Enriqueta Pujol-Ribera

**Affiliations:** 1 Divisió d'Avaluació, Servei Català de la Salut, Barcelona, Spain; 2 Institut d'Investigació en Atenció Primària Jordi Gol, Barcelona, Spain

## Abstract

**Background:**

Validated instruments are needed to evaluate the programmatic impact of Evidence Based Practice (EBP) training and to document the competence of individual trainees. This study aimed to translate the Fresno test into Spanish and subsequently validate it, in order to ensure the equivalence of the Spanish version against the original English version.

**Methods:**

Before and after study performed between October 2007 and June 2008. Three groups of participants: (a) Mentors of family medicine residents (expert group) (n = 56); (b) Family medicine physicians (intermediate experience group) (n = 17); (c) Family medicine residents (novice group) (n = 202); Medical residents attended an EBP course, and two sets of the test were administered before and after the course. The Fresno test is a performance based measure for use in medical education that assesses EBP skills. The outcome measures were: inter-rater and intra-rater reliability, internal consistency, item analyses, construct validity, feasibility of administration, and responsiveness.

**Results:**

Inter-rater correlations were 0.95 and 0.85 in the pre-test and the post-test respectively. The overall intra-rater reliability was 0.71 and 0.81 in the pre-test and post-test questionnaire, respectively. Cronbach's alpha was 0.88 and 0.77, respectively. 152 residents (75.2%) returned both sets of the questionnaire. The observed effect size for the residents was 1.77 (CI 95%: 1.57-1.95), the standardised response mean was 1.65 (CI 95%:1.47-1.82).

**Conclusions:**

The Spanish version of the Fresno test is a useful tool in assessing the knowledge and skills of EBP in Spanish-speaking residents of Family Medicine.

## Background

Educators implementing Evidence Based Practice (EBP) training need instruments to evaluate the programmatic impact of new curricula and to document the competence of individual trainees. Several systematic reviews have examined the instruments used to evaluate the effectiveness of educational programs. [1,2] Shaneyfelt et al. reviewed the available EBP teaching instrument methods, including 115 studies that represented 104 unique instruments[2]. The authors identified high-quality instruments for evaluating the EBP competence of individual trainees and for determining the effectiveness of EBP curricula. High quality instruments are distinguished by the ability to discriminate between different levels of expertise or performance and are suited to document the competence of individual trainees. Furthermore, the robust psychometric properties, in general, support their use in formative or summative evaluations. They are supported by established inter-rater reliability (if applicable), objective (non-self-reported) outcome measures, and multiple (≥ 3) types of established validity evidence (including evidence of discriminative validity).

Within high quality instruments, the Fresno test evaluates the most EBP steps [3]. It begins with the presentation of two clinical scenarios. Seven short answer questions about the clinical scenarios require the candidate to formulate a focused question, identify the most appropriate research design for answering the question, show knowledge of electronic database searching, identify issues important for determining the relevance and validity of a given research article, and discuss the magnitude and importance of research findings. A series of 5 questions requiring calculations and filling in the blank are asked as well. Open ended questions are scored with standardised grading rubrics.

Reliability and validity are context-specific attributes rather than fixed properties and therefore must be assessed in relation to the specific population and context [4]. An instrument that has demonstrated satisfactory measurement properties in one population is not necessarily appropriate for use in other populations [5]. The validation of the Fresno questionnaire into different languages, professional groups, and cultural settings, will enable the generalisability of the test, as well as allowing comparisons between countries and the evaluation of different teaching methods.

The aim of the current study was the translation of the Fresno questionnaire into Spanish and its subsequent validation to ensure the equivalence of the Spanish version against the original English version.

## Methods

The study was comprised of two stages: translation of the instrument into Spanish and its subsequent validation. More information has been previously published in the study protocol [6].

### Participants and study description

The instrument was validated by administering it to three groups: (a) The first group included mentors of family medicine residents with formal methodological training in EBP. They belong to a network of physicians who are regularly engaged in the design and conduct of clinical trials in primary care (expert group) (n = 56). The network holds its annual conference in November. (b) The second group included Family Medicine and Community Medicine physicians (intermediate group) (n = 17). (c) The third group included family medicine residents in the first and second year of the Family Medicine training programme in Catalonia, Spain, before they had had any formal training in EBP in their residence program (novice group) (n = 202). [6]

The following variables were recorded for each learner: age, sex, year of graduation from medical school, courses in EBP completed prior to the educational intervention and time required to fill-in the test. In order to assess the feasibility of the test, the time required to complete the test was recorded.

The setting of the study was a Primary Care Teaching Unit (PCTU). The family medical residents and physicians were enrolled in a PCTU.

### Scoring the test

The authors developed scoring criteria based on predicted responses, as well as on their expert opinion about the elements of an ideal answer. The total test score is the sum of points for all items, and the maximum possible score is 212 points. In order to assess inter-rater reliability two scorers independently scored a random sample of 40 questionnaires. They were blind to the identity of the participants. Previously they did a scoring pilot test with ten tests to agree on the scoring methodology.

Following the criteria described by Ramos et al [3], for each item, limited performance in each category would result in a score of 8. We therefore considered any total less than 8 for a question as "not evident". A score of 8-15 was defined as a limited response, 16-23 as a strong response, and 24 as an excellent response.

### Translation of the Fresno test into Spanish

First, an e-mail was sent to the original developers of the Fresno test asking permission to proceed with the use and translation of the tool for research purposes.

**Figure 1:**
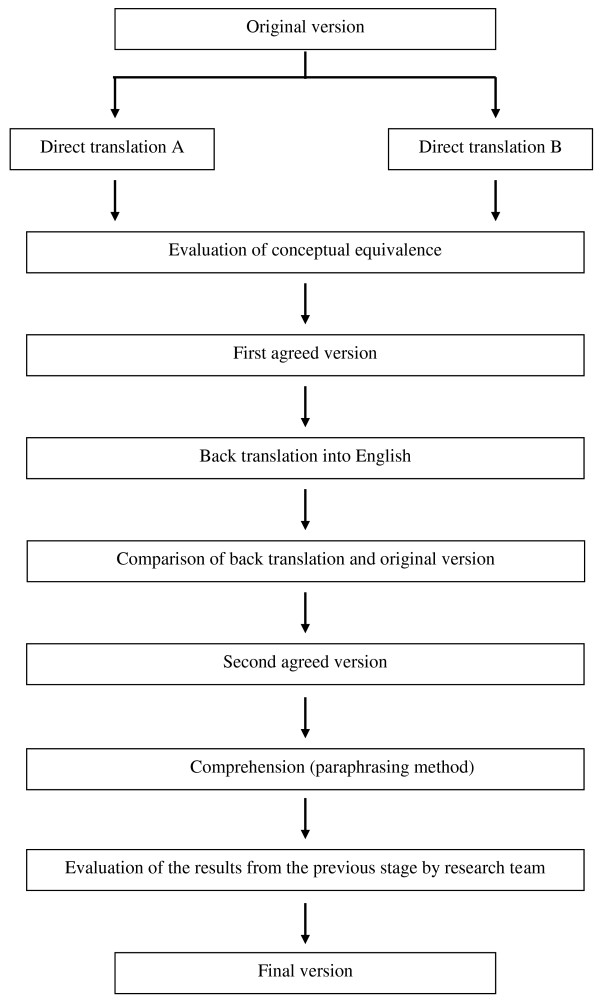
**Translation process of the Fresno questionnaire into Spanish**.

The test was translated and back-translated according to guidelines for questionnaire adaptation in order to achieve the highest possible content validity [7,8] (Figure 1).

The original English version of the Fresno questionnaire was translated into Spanish independently by two bilingual translators. A team made up of EBP experts, specialists in Family and Community Medicine and specialists in Preventive Medicine and Public Health reviewed the translations. Based on the translations and the comments raised by the research team, an agreed Spanish version of the questionnaire was obtained. The next step was to back-translate the agreed Spanish version to make sure that it was conceptually equivalent to the original version. Subsequently, the research team, with assistance from all the translators, compared the back-translation with the original version in order to identify any questions that were not equivalent or which may have been problematic.

Once the agreed Spanish version was obtained, a series of individual interviews were conducted with residents and specialists in Family and Community Medicine to assess the comprehension of the questionnaire (cognitive debriefing). The interviews were evaluated based on the degree of understanding of the items and the ease or difficulty in filling-in the questionnaire. The interviews were conducted with 5 residents and 5 specialists in Family and Community Medicine. The paraphrasing method was used in the interviews, in which the participants rephrased the items that presented the greatest difficulty.

Finally, the research team met to evaluate the results of the questionnaire comprehension tests and to obtain the final version of the pre-test [6].

### EBP course and administration test

The EBP course is made up of four intensive and interactive half-day sessions that are designed to develop the knowledge and skills required to practice evidence-based care. The course was modelled after the steps of EBP [9]. Educational activities were performed between October 2007 and June 2008. The course is obligatory for the residents in this specialty. Tests were administrated before the start of the EBP course (pre-test) and on the last day of the course (post-test). In both cases, the tests were administered during the class with the investigators present. All the learners were explicitly informed about the research nature of the test and that participation was voluntary.

The expert group did not attend the EBP course and was used to assess the equivalence between the pre-test and post-test In order to assess the equivalence of the two sets of the questionnaire (pre-test and post-test), both sets were randomly assigned among the folders distributed to the expert participants when they attended the annual conference in November. The same process was used in the intermediate group. Equivalence of the two sets was determined by calculating the mean difference between them using a t-test [6].

### Psychometric measurements and statistical analysis

Descriptive statistics were used to summarize demographic characteristics and scores on items of the test.

Several cases were excluded from the analysis; the learners who did not fill-in the pre-test and those who failed to answer the post-test were not taken into account for the responsiveness analysis, as well as those learners who did not attend at least three half-day sessions of the EBP course.

To assess the construct validity of the test, the expertise of the participants was used as an important indicator for knowledge and skills in EBP. The scores achieved by the experts were compared with the post-test scores obtained by the novice group and the intermediate experience group, using an analysis of variance. If a significant difference was found, a post hoc analysis by Scheffé's method was added.

Cronbach's alpha coefficient was used to evaluate the internal consistency. Pearson's correlation coefficient was also calculated to assess the corrected item total correlation to identify items contributing to a low reliability [4]. Inter-rater reliability and intra-rater reliability were assessed using Kappa coefficient for qualitative items, and intra-class correlation coefficient (ICC) was used for quantitative items and the overall score [4]. As in the original version, we assigned a cut off for a "passing" answer in order to assess the difficulty of items in the test. We used the same definition as in the original test and set this cut off as the midpoint of the strong response category [3]. Item discrimination was calculated by ranking the participants according to total score and then selecting the top quartile and the lowest quartile. For each item, the percentage of participants in the upper and lower groups that answered correctly was calculated. The difference was one measure of item discrimination.

Effect size and standardised response mean were the responsiveness statistics that were used in the analysis. The effect size is the difference between the mean baseline and follow-up scores of the measure, divided by the standard deviations from the baseline scores. Positive values reflect (standardized) improvements in the number of standard deviations from the baseline scores (ES) [10]. The effect size was defined as "small" (E-S < 0.2), "small to moderate" (E-S between 0.2 and 0.5), "moderate to large" (E-S between 0.51 and 0.79), "large" (E-S > 0.79) [11]. The Standardised Response Mean (SRM) was calculated as the mean change in scores divided by the standard deviation of these changes. Positive values reflect (standardized) improvements in the number of standard deviations from the score differences [12].

Differences between the post-intervention scores and the pre-intervention scores were analysed using a paired t-test. Differences between the percentages of passing scores pre and post intervention by question were examined using a Chi-square test.

The questionnaires were completed on paper, and all data were entered electronically at the end of the course. Statistical analyses were conducted by using Stata software version 9.0 (STATA Corp, College Station, TX) and with SPSS software version 15.0 for Windows (SPSS, Chicago, IL, USA).

## Results

Most items in the questionnaire were translated into Spanish without difficulty. Based on the responses from the cognitive debriefing interviews, participants found the Spanish version to be clear, unambiguous, comprehensive and easy to complete. Discrepancies were solved by consensus. No major changes were required as a result of the debriefing exercise. The exception was the word "evidence" which has a slightly different meaning in Spanish. The translators used the word "*prueba*" as a more accurate translation for evidence. Nevertheless, since the word "*evidencia*" has been extensively used in Spanish (i.e. the journal "Evidence Based Medicine" has been translated as "*Medicina Basada en la Evidencia"*), participants found it more appropriate to use the word "*evidencia*".

275 people took part in the study: 56 experts in evidence based medicine, 17 participants in the intermediate experience group, and 202 residents in their first or second year of training. 190 of 202 (94.1%) residents completed the pre-test, and 158 (78.2%) residents returned the post-questionnaire. Overall, 152 residents (75.2%) returned both sets of the questionnaire. The main reasons for partial completion were failure to submit the questionnaire after the course (n = 18), failure to participate in the course (n = 16), failure to submit the pre-test (n = 8) and failure of identification (n = 8). Only 44 residents (23.2%) stated that they had formal, structured training in evidence based practice prior to the course.

The mean difference of scores between pre-test and post-test obtained by experts was 2.1 (CI 95%: -10.4 to 14.7).

Since both sets of the questionnaire were equivalent, the entire group of experts and the entire group of participants with intermediate experience were used for the validation of the pre-test and post-test. Therefore, 263 participants took part in the validation of the pre-test questionnaire, and 231 took part in the validation of the post-test questionnaire. Finally, the responsiveness was examined in the group of residents who returned both sets of questionnaires (n = 152).

The average age of the participants was 31 years (SD = 8.0) and 76.3% were women. Only 44 residents (23.2%) stated that they had formal, structured training in evidence based practice prior to the course.

The test showed a high overall internal consistency. The alpha coefficient in the pre-test was 0.88, whereas in the post-test it was 0.77. The corrected item-total correlation coefficients (table 1) were generally between 0.36 for question 1 (formulating a question) and 0.67 for question 3 (searching the evidence) in the pre-test questionnaire. In the post-test questionnaire, the coefficients ranged from 0.25 (question 11: best study design, diagnosis) to 0.52 (question 5: relevance).

**Table 1:** Item -to-total correlations analyses of the Spanish version of the Fresno test

Question	Pre-test	Post-test	Original version
Q1 Formulate Question	0.36	0.35	0.67
Q2 Sources of information	0.41	0.45	0.47
Q3 Search	0.67	0.43	0.58
Q4 Study Design	0.55	0.48	0.71
Q5 Relevance	0.61	0.52	0.50
Q6 Internal validity	0.50	0.48	0.61
Q7 Magnitude of effect	0.58	0.49	0.75
Q8 Sensitivity	0.51	0.37	0.51
Specificity	0.45	0.36	0.65
Positive Predictive Value	0.46	0.36	0.56
Negative Predictive Value	0.48	0.37	0.58
Positive Likelihood Ratio	0.35	0.37	0.62
Q9 Absolute Risk Reduction	0.59	0.44	0.63
Relative Risk Reduction	0.66	0.41	0.60
Number Needed to Treat	0.56	0.33	0.66
Q10 Confidence Interval	0.59	0.46	0.75
Q11 Best Study Design, Diagnosis	0.54	0.25	0.56
Q12 Best Study Design, Prognosis	0.59	0.37	0.53

Q: Question

The overall inter-rater reliability was 0.95 and 0.85 in the pre-test and post-test questionnaire, respectively (table 2). The overall intra-rater reliability was 0.71 and 0.81 in the pre-test and post-test questionnaire, respectively. Results ranged from moderate to good depending on the test question being scored. In the pre-test, the lowest intra-rater reliability was found in question six and in the post-test was found in question five (table 3).

**Table 2:** Inter-rater reliability of the Spanish version of the Fresno test

Question	Pre-test	Post-test	Original Version
Q1 Formulate Question	0.97	0.86	0.89
Q2 Sources of information	0.95	0.87	0.94
Q3 Search	0.92	0.92	0.92
Q4 Study Design	0.87	0.73	0.96
Q5 Relevance	0.88	0.90	0.84
Q6 Internal validity	0.89	0.76	0.72
Q7 Magnitude of effect	0.99	0.79	0.84
Q1 thru Q7	0.95	0.85	0.97

Q: Question

**Table 3:** Intra-rater reliability of the Spanish version of the Fresno test

Question	Pre-test	Post-test
Q1 Formulate Question	0.78	0.90
Q2 Sources of information	0.65	0.74
Q3 Search	0.65	0.83
Q4 Study Design	0.71	0.84
Q5 Relevance	0.46	0.34
Q6 Internal validity	0.44	0.66
Q7 Magnitude of effect	0.72	0.85
Q1 thru Q7	0.71	0.81

Q: Question

Every item had a positive value and helped to distinguish between different levels of expertise (table 4). In the post-test questionnaire, only question 7 (magnitude of effect) had a value below the pre-defined value of 0.20. In contrast in the pre-test four questions did not achieve the pre-defined value of 0.20. The items ranged in difficulty from moderate to difficult (table 5).

**Table 4:** Item Analysis: Item Discrimination of the Spanish version of the Fresno test

Question	Pre-test	Post-test	Original version
Q1 Formulate Question	0.32	0.29	0.73
Q2 Sources of information	0.15	0.22	0.41
Q3 Search	0.16	0.29	0.59
Q4 Study Design	0.36	0.46	0.68
Q5 Relevance	0.24	0.33	0.45
Q6 Internal validity	0.24	0.59	0.68
Q7 Magnitude of effect	0.07	0.19	0.86
Q8 Sensitivity	0.47	0.82	0.64
Specificity	0.44	0.83	0.86
Positive Predictive Value	0.42	0.80	0.73
Negative Predictive Value	0.36	0.82	0.77
Positive Likelihood Ratio	0.14	0.80	0.73
Q9 Absolute Risk Reduction	0.28	0.48	0.77
Relative Risk Reduction	0.21	0.83	0.68
Number Needed to Treat	0.31	0.67	0.77
Q10 Confidence Interval	0.24	0.62	0.82
Q11 Best Study Design, Diagnosis	0.17	0.54	0.55
Q12 Best Study Design, Prognosis	0.20	0.68	0.64

Q: Question.

Values represent the difference in proportions of test takers answering correctly between those scoring in the upper 27% on total score and those scoring in the lower 27%.

**Table 5:** Item Analysis: Item Difficulty of the Spanish version of the Fresno test

Question	Pre-test N =	Post-test N =	Original version Novice	Original Version Expert
Q1 Formulate Question	0.29	0.33	0.27	0.80
Q2 Sources of information	0.20	0.21	0.61	0.75
Q3 Search	0.15	0.35	0.17	0.64
Q4 Study Design	0.38	0.51	0.44	0.80
Q5 Relevance	0.29	0.38	0.30	0.37
Q6 Internal validity	0.21	0.58	0.56	0.91
Q7 Magnitude of effect	0.10	0.20	0.12	0.58
Q8 Sensitivity	0.54	0.84	0.60	0.84
Specificity	0.44	0.77	0.33	0.76
Positive Predictive Value	0.44	0.79	0.40	0.71
Negative Predictive Value	0.39	0.78	0.35	0.66
Positive Likelihood Ratio	0.17	0.46	0.15	0.58
Q9 Absolute Risk Reduction	0.40	0.84	0.33	0.87
Relative Risk Reduction	0.24	0.66	0.10	0.76
Number Needed to Treat	0.25	0.62	0.30	0.87
Q10 Confidence Interval	0.29	0.56	0.26	0.86
Q11 Best Study Design, Diagnosis	0.21	0.78	0.10	0.39
Q12 Best Study Design, Prognosis	0.24	0.68	0.41	0.83

Values represent proportion of scores that exceeded "passing" for each item.

Participants with intermediate experience scored moderately on the questionnaire administered before the course [mean score 110.4 (SD = 11.5)], whereas experts scored better [149.8 (SD = 23.2)] and novices poorly [60.4 (SD = 25.0)] (p < 0.001). Residents who self-reported former training in evidence based practice had higher scores than the rest of the residents [75.9 (SD = 29.2) vs 57.6 (SD = 23.1): CI 95%: 9.1-25.6; p < 0.0001].

Responsiveness was examined only in those residents who returned both versions of the questionnaire (n = 152). On the pre-test survey, the average score for residents was 63.9 (SD = 24.3). On the post-test survey, the average score was 111.6 (SD = 30.4). Using a difference score as the criterion in a paired t-test, the mean difference between pre-test and post-test was 47.7, a statistically significant result (95% CI: 42.8-52.5; *p *< 0.0001). In percentage, the residents gained on average 19.7% of score of the total possible score. In the subgroup of residents with former training in EBP the difference before and after the intervention was: 45.8 (CI 95%: 35.5-56.2; p < 0.0001)

The observed effect size for the residents was 1.77 (CI 95%: 1.57-1.95), and the standardised response mean was 1.65 (CI 95%:1.47-1.82). In the subgroup of residents with former training, the responsiveness indices were similar to those found in the total group of residents (Table 6).

**Table 6:** Responsiveness indices by group of the Spanish version of the Fresno test

	Total group of residents (CI 95%)	Residents with former training (CI 95%)
Effect Size	1.77 (1.57-1.95)	1.78 (1.38-2.17)
Standardised Response Mean	1.65 (1.47-1.82)	1.60 (1.24-1.96)

The mean time required for filling-in the test was 40 minutes (SD = 8.1) for novices and the group with intermediate experience. The time required for experts was 35 minutes (SD = 8.7). The mean time required for scoring the test was 12 minutes (SD = 3).

## Discussion

This study demonstrated the utility of the Spanish version of the Fresno test to evaluate the improvement in knowledge and skills after a formal training in EBP. The questionnaire effectively differentiated various levels of expertise in the participants, the estimated coefficients of internal consistency showed high reliability and the responsiveness observed was quite high by both methods of analysis. Similar to the original version, neither floor nor ceiling effect was evident in the Spanish language version. This means that both novices and experienced practitioners can be assessed.

Cronbach's alpha, which measures the overall correlation between items within a scale, was 0.88 in the pre-test, the same value observed in the original version and above the widely accepted value of 0.70 [4]. The post-test Cronbach's alpha was 0.77. This result indicates a good internal consistency, particularly given that in order to achieve a high discriminatory power, a scale measuring knowledge must include easy items as well as difficult items. This tends to decrease the internal consistency of the scale.

Construct validity involves comparisons between measures and examines the logical relations that should exist between a measure and characteristics of patients and patient groups. The first step in construct validation is to establish a model or theoretical framework that represents an understanding of what investigators are trying to measure [12]. In the present study we have tested two hypotheses. First, that the test should discriminate the expert group of participants from novices and those with intermediate experience. Second, that those residents with former instruction in EBP should have higher scores than the rest of residents. The results confirmed both hypotheses, strengthening the discriminative validity of the test.

Item difficulty is important because it reveals whether an item is too easy or too hard. In either case, the item may add to the unreliability of the test because it does not aid in differentiating between participants. The optimal item difficulty depends on the question-type. Nevertheless, scores per question by course participants should not fall below 0.1 or go above 0.9, as scores outside these parameters do not tend to provide additional information to distinguish the more knowledgeable participants from the less knowledgeable ones. Such items should either be revised or replaced. In our study we found a wide range of item difficulties which allows the test to be used with both expert and novice groups. Differences between our study and the original study may be attributed to the different backgrounds of EBP knowledge and skills. Whereas in our population the ratio of family medicine residents to self-identified experts in EBP was 3.5, in the original Fresno test this ratio was 0.8.

The responsiveness of the Spanish version of the Fresno was observed to be quite high by both methods of analysis. The values of the responsiveness indices were 1.77 and 1.65 for the effect size and the standardized response mean, respectively. Similar results were found in the subgroup of residents with prior training in EBP. These results provide evidence that the test is sensitive to educational change in the context of a before-after educational trial.

The differences between indices in this study, although not large, can be explained by the fact that different methods have different goals. While the two indices use the mean change of the score over time by subtracting the baseline score from the data obtained after the educational intervention, there are significant differences in how the standard deviations or variability in the data is used in the calculation. Therefore, it is possible that significant differences exist in the variability in the selected subgroups, resulting in differences in the perceived responsiveness of the measure depending upon the responsiveness index chosen. Statistics such as the SMR that use the standard deviation of the change in score are meant to show statistically significant changes, whereas effect sizes that use the standard deviation of the baseline score are meant to quantify the amount of changes [13].

The complexity and length of an instrument may jeopardise the conduct of research and disrupt educational efforts. The time required for filling-in the test is 40 minutes, which could be a major shortcoming for its use in routine practice. Furthermore, the time needed to score the questionnaire is longer than the time required to score a similar questionnaire with multiple choice questions. Staff training needs must be considered before undertaking administration. The results of this study indicate that examiners need formal structured scoring training to achieve good intra-rater and inter-rater reliability. Finally, staff attitudes and acceptance of instruments can make a substantial difference to respondent acceptability.

### Strengths and limitations

Several strengths of this study should be highlighted. First of all, validation of a questionnaire is an ongoing process that continues with the repeated use of an instrument and does not end when the first study with data concerning validity is published. The validation of the Fresno questionnaire into Spanish, in a population with a different background of experience in EBP than the population recruited by Ramos et al [3], will enable the generalisability of the test, as well as allow comparisons between countries and the evaluation of different teaching methods. The test was validated in a sample with a wider range of experience in EBP than the sample studied by Ramos et al. Secondly, the current study provides an estimation of the amount of change in knowledge and skills, which was not provided in the original version. This will help to estimate the required sample size of clinical trials and the effectiveness of an educational intervention. Thirdly, this study was performed in routine conditions, with multiple lecturers who were advised not to modify their sessions with a view to coaching for the test. When several lecturers are involved there is always some difficulty in standardising the intervention. Lack of standardisation will inflate error variance and decrease the chance of obtaining true differences. On the other hand, lack of standardisation is typical for pragmatic trials and reflects real situations [14]. Finally, we have assessed the feasibility of the instrument.

Several potential limitations should be considered in the results and interpretation of this study. Although the test was validated in a sample with a wider range of experience in EBP than the sample studied by Ramos et al [3], there was a predominance of those considered novices, and the percentage of general practitioners with intermediate experience was low (5%). This could be considered a methodological limitation since a different structure of the sample could have led to different psychometric indices. The differences between the pre-test and the post-test questionnaire could be explained by the improvement in knowledge among residents. Furthermore, 44 residents were missing either the pre-test or post-test. It is possible that restricting the sample to subjects who had completed responses on all the measures may have changed the properties of the test. Those who did not return their test were likely to have been less engaged and possibly less knowledgeable and confident than those who responded.

Although the overall intra and inter-rater agreement was high in the current study, the reliability of the scoring process was less than 100%. Hence, there may be some misclassification during the scoring process. This in turn may result in a bias towards the null hypothesis when the test is used in educational intervention studies. Furthermore, the agreement between individual raters appears to be substantially higher than the agreement between different assessments by the same individual. One possible reason is that the intra-rater assessments were done on both versions of the test at the beginning of the study, whereas the inter-rater assessments were assessed in the middle of the scoring process after having scored more than 100 tests. This suggests that the raters improved substantially during the course of the study, to the point that, by the end, inter-rater agreements paradoxically exceeded the original intra-rater agreement. This result has several possible implications. First, formal training of raters is needed before using the Fresno Test as an evaluative instrument. Second, some of the changes in knowledge and skills reported in this study may reflect measurement error because of less than perfect intra-rater reliability in some questions. Third, further refinement of the Fresno Test scoring system is indicated. We also suggest that, when using the Fresno Test, it is important to measure not only inter-rater reliability but also intra-rater agreement. Intra-rater reliability was not assessed by Ramos et al [3]; therefore, we cannot compare the current results with those in the original study. However, it was assessed by McCluskey, who observed an excellent agreement between raters [15].

Finally, for logistical reasons, only residents and Family Medicine mentors were included in the validation study. While it could be suggested that more similarities than differences exist between the different groups of medical residents with regard to EBP issues, the use of Fresno Test in a population including residents from other specialties would require testing for validity and reliability in the specific resident group. Levels of inter-rater reliability, internal consistency and discrimination are intimately dependent on the population which has taken the test; therefore, it cannot be assumed that any of these key attributes would be maintained in subsequent studies with a population that has a different EBP background.

We may have varying degrees of confidence that an instrument is really measuring what it is supposed to measure. The more frequently an instrument is used, and the more situations in which it performs as expected, the greater the confidence in its validity. In this sense, there are a number of further developments of the Fresno test that are required. The development of new clinical scenarios and new sets of numerical examples, as well as the administration of the test, is a time-consuming task that could lead to the use of more simple and quick-to-administer instruments, such as multiple choice questionnaires. Future research could include the development and validation of a shorter version of the test using the long version as a gold standard.

While the intra-rater reliability of the Spanish version of the Fresno test overall score is good, further work is required to improve the scoring system and reliability of a subset of questions. It is important to ascertain if the effect of ongoing training and the increasing familiarization with the nuances of the scoring process over time increase intra-rater reliability. It would also be an indirect measure of how much training is needed before using the Fresno Test in practice.

## Conclusions

The findings of this study indicate that the Spanish language version of the Fresno test is a comprehensible and appropriate instrument. as well as a reliable tool in assessing the knowledge and skills in EBP in Spanish-speaking Family Medicine residents. The Fresno Test is also a good evaluative instrument since it is able to measure how much knowledge and skills have changed during a period of time after an educational intervention. Therefore, it can be used as an outcome variable in randomised controlled trials.

## Competing interests

The authors declare that they have no competing interests.

## Authors' contributions

JAP and GFM are the principal investigators responsible for the conception of the project and drafting the manuscript. JJV and GFM will be in charge of the statistical analyses. All authors have contributed to the description of the background and general design. All authors have read and approved the final manuscript.

## Pre-publication history

The pre-publication history for this paper can be accessed here:

http://www.biomedcentral.com/1472-6920/10/45/prepub
